# Suspected Macrocreatine Kinase Type 1 in an Asymptomatic Young Cat

**DOI:** 10.1155/crve/3714485

**Published:** 2026-05-30

**Authors:** Satoshi Kambayashi, Kenji Baba, Yutaro Ide, Shoma Nishibori, Takanori Ando, Hiroki Ikezawa

**Affiliations:** ^1^ Department of Veterinary Internal Medicine, Joint Faculty of Veterinary Medicine, Yamaguchi University, Yamaguchi, Japan, yamaguchi-u.ac.jp; ^2^ Department of Veterinary Clinical Pathology, Joint Faculty of Veterinary Medicine, Yamaguchi University, Yamaguchi, Japan, yamaguchi-u.ac.jp; ^3^ Heart-Will Animal Hospital, Fukuoka, Japan; ^4^ Animal Medical Technology Co., Ltd., Nagoya, Aichi, Japan

## Abstract

A 1‐year‐old castrated male Japanese domestic cat was referred for persistently elevated serum creatine kinase (CK) level (> 2000 U/L), which was detected incidentally. The cat showed no clinical signs, with no abnormalities on physical examination, radiography, and ultrasonography. Serum CK isoenzyme analysis by agarose gel electrophoresis revealed an atypical high distribution of CK‐MB (90%). However, CK‐MB–specific quantification by chemiluminescent immunoassay showed significantly low concentration (< 0.1 ng/mL). Based on these observations, we suspected macro‐CK type 1. Serum CK is used as a biomarker of muscle injury in both humans and other mammals. Macro‐CK type 1, a complex of CK and immunoglobulin, has been recognized as the cause of persistently elevated CK levels with or without disease in human medicine. On the other hand, reports of macro‐CK are scarce in veterinary medicine. Based on this case, when persistently elevated CK was detected in clinically healthy cats, this disease should be included as a differential diagnosis. Further studies are required to establish the diagnosis of macro‐CK type 1 in cats.

## 1. Introduction

Creatine kinase (CK) is an enzyme that catalyzes the reaction converting creatine and adenosine triphosphate (ATP) into phosphocreatine and adenosine diphosphate (ADP). Since CK is abundant in muscle tissue, it has been clinically applied as a marker of muscle injury such as rhabdomyolysis or myocardial infarction [1]. CK is a dimer composed of two subunits, designated B and M [2]. Isoenzyme analysis indicates that CK‐MM, derived from skeletal muscle, is the major compartment in serum CK, CK‐BB is brain‐derived, and CK‐MB is myocardial‐derived [3]. In human medicine, it is well known that there are two additional forms of CK, termed macro‐CK type 1 and type 2, in addition to the three conventional CK isoenzymes. Macro‐CK type 1, a complex of CK enzyme and immunoglobulin via antigen–antibody reaction, is known to be associated with diseases such as autoimmune disorders [4], hypothyroidism [5], and neoplasms [6]. However, it may also occur in otherwise healthy individuals without any underlying disease [7]. Macro‐CK type 2 is formed from mitochondrial CK and associated with tumor [8, 9].

In cats, one study has reported macro‐CK [10], which demonstrated increased macro‐CK type 1 in both cats with and without central nervous system signs. Here, we report that suspected macro‐CK type 1 is characterized by continuous CK elevation in a young cat with no clinical signs.

## 2. Case Presentation

A 1‐year‐old castrated male Japanese domestic cat weighing 3.98 kg was referred to the Yamaguchi University Animal Medical Center with persistently elevated CK levels, which were incidentally detected. Two months prior to the presentation, the cat underwent a biochemical examination at a primary animal hospital (T.A.) and revealed markedly elevated CK (> 2000 U/L). Subsequent tests performed every 2–4 weeks showed no decrease. Given the persistent elevation of CK, detailed examinations of the cardiovascular system were performed. However, no significant abnormalities were observed on the thoracic radiograph, and the echocardiogram showed no findings suggesting heart failure, cardiomyopathy, or congenital heart defects. Cardiac troponin I was also measured, and the result was 0.053 ng/mL, which was within the reference range (< 0.121 ng/mL; FUJIFILM, Tokyo, Japan).

The patient was kept indoors and had no history of trauma, intoxication, and subcutaneous/intramuscular injections. On physical examination, the cat showed no abnormalities, including muscle atrophy or decreased motor function, and no pain was elicited. Thoracic and abdominal radiographs revealed no abnormalities, and the musculature of the trunk and limbs appeared normal and symmetrical. As shown in Table 1, biochemical examination indicated elevation of CK level (2835 U/L), while all other biomarkers, including aspartate aminotransferase (AST) and lactate dehydrogenase (LDH), were within their respective reference range (Dri‐chem NX600, FUJIFILM). Feline immunodeficiency virus antibodies and feline leukemia virus antigens were tested using a rapid assay kit (IDEXX Laboratories Japan, Tokyo, Japan), and both were negative.

**Table 1:** Complete blood count and biochemical examination.

**Hematology**	**Value**	**Unit**	**Reference interval**

RBC	12.9	10^6^/*μ*L	5.0–10.0
HCT	46.1	%	24.0–45.0
HGB	14.7	g/dL	5.5–19.5
MCV	36	fL	39–55
MCH	11	pg	13–17
MCHC	31.9	g/dL	30–36
WBC	6080	*μ*L	5500–19,500
Neutrophils	4270		2000–8000
Lymphocytes	1610		1000–5000
Eosinophils	180		0–1200
Monocytes	20		0–1000
Platelets	226	10^3^/*μ*L	100–400

**Serum chemistry**	**Value**	**Unit**	**Reference interval**

Blood urea nitrogen (BUN)	24.1	mg/dL	17.6–32.8
Creatinine	1.76	mg/dL	0.90–2.10
Alanine transaminase (ALT)	78	U/L	22–84
Aspartate transaminase (AST)	52	U/L	18–51
Alkaline phosphatase (ALP)	29	U/L	−58
Lactate dehydrogenase (LDH)	99	U/L	−187
Creatine kinase (CK)	2835	U/L	87–309
Cholesterol	142	mg/dL	95–259
Glucose	108	mg/dL	71–148
Na	156	mEq/L	147–156
K	3.7	mEq/L	3.4–4.6
Cl	118	mEq/L	107–120
Serum amiroid A	< 3.75	*μ*g/mL	−5.49

**Figure 1:**
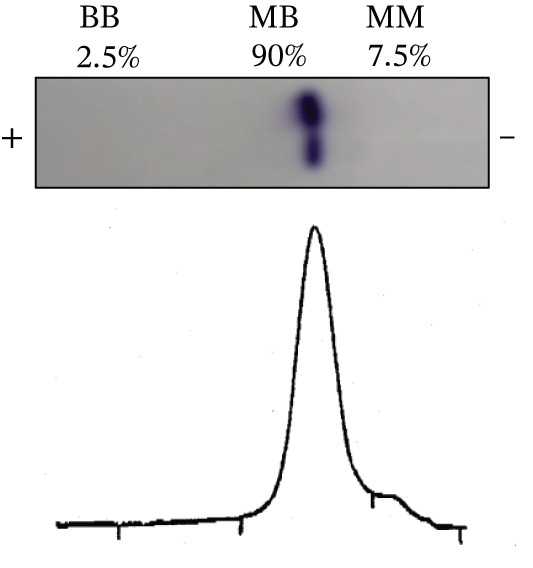
Image and analytical results of CK macroenzymes by agarose gel electrophoresis. The values indicate the band intensities mechanically quantified.

For CK isoenzyme analysis, the patient′s serum was examined by agarose gel electrophoresis resulting in proportions of CK‐BB, CK‐MB, and CK‐MM of 2.5%, 90%, 7.5%, respectively (Figure 1). However, dimerized CK in mammalian myocardium consists of around 70% of CK‐MM and 25%–30% of CK‐MB [11]. Therefore, the marked isolated increase of CK‐MB isoenzyme suggests an assay artifact rather than myocardial injury. Subsequently, CK‐MB–specific measurement was performed by chemiluminescent immunoassay (CLIA, ARCHITECT *i*2000SR, Abbot Japan LLC, Tokyo, Japan), revealing a concentration below 0.1 ng/mL, thereby excluding myocardial injury as the cause of the CK elevation.

Finally, it was speculated that the cause of the falsely elevated CK by blood chemistry tests and CK‐MB by CK isoenzyme analysis was macro‐CK type 1, which was also observed in young, healthy humans. The cat received no specific treatment and has remained clinically asymptomatic for 332 days at the most recent follow‐up.

## 3. Discussion

Macro‐CK type 1 is well recognized in human medicine but rarely reported in veterinary medicine. Macro‐CK type 1, typically formed from a complex of CK‐BB and immunoglobulin G [12], has a high molecular weight that prolongs its half‐life in blood and consequently leads to elevated CK levels on biochemical examination. This typically results in sustained elevations in CK level that plateau at a certain level, rather than progressively increasing over time. In humans, macro‐CK type 1 is generally associated with a favorable prognosis when asymptomatic, and its long‐term clinical implications remain uncertain. Elevations of macro‐CK type 1 have also been described in association with autoimmune myositis or other myopathies [4, 8].

Only a few studies have reported macro‐CK in dogs and cats, and the relationship between macro‐CK and diseases was evaluated in neurologic disorders [10, 13]. In those studies, macro‐CK type 1 in dogs and cats has been speculated to be associated with inflammation, epilepsy, and intracranial tumors. Additionally, both cats referred to feline infectious peritonitis with neurological signs and healthy cats showed significantly higher macro‐CK, but not in dogs. Therefore, it is postulated that a considerable proportion of cats may harbor asymptomatic macro‐CK type 1. Persistent CK elevation in an otherwise healthy cat may lead clinicians to suspect muscular or cardiac disease, potentially prompting unnecessary diagnostic procedures or even unwarranted treatments; therefore, recognizing macro‐CK type 1 as a benign differential diagnosis is important.

In this case, the cat presented no significant clinical signs, no abnormalities on blood analysis and diagnostic imaging, except for continuously elevated CK level. Furthermore, an extremely high level of CK‐MB proportion has been observed by electrophoresis, while CK‐MB–specific quantification showed a low concentration of CK‐MB. Based on these findings, we considered this case to represent macro‐CK type 1. However, since there is no established diagnostic definition for this disease in veterinary medicine, finally, we diagnosed this case as suspected macro‐CK type 1. Given that macro‐CK type 1 has been documented in association with various diseases, particularly muscular disorders, additional diagnostic investigations such as electromyography, computed tomography (CT), or muscle biopsy would be indicated. Nevertheless, these procedures could not be performed due to the owner′s circumstances. Therefore, continued monitoring is required to detect potential signs of disease in this case.

## 4. Limitations

There are some limitations in this report. First, since feline CK isoenzyme analysis is not well established, it is unclear whether the isolated CK isoenzymes truly reflect the actual condition. In this report, we sent serum for a commercially available external laboratory test examining CK isoenzyme, which was analyzed by using gel electrophoresis. However, this test is based on the human system and has not been optimized for cats. Furthermore, the classification of CK isoenzymes is performed by reference to electrophoretic pattern established in humans. The same applies to the quantification of CK‐MB using antibody against human CK. Theoretically, human and feline CK‐M and CK‐B are well‐conserved proteins with amino acid sequence identities of 98% and 98%, respectively. Therefore, CK‐MB–specific measurement in cats could also be feasible using the human system. However, the establishment of a feline‐specific assay would be preferable. Second, detailed assessments for muscles such as CT and/or electromyography to evaluate localized myopathies were not conducted due to economic reasons of the owner in this case. Given the absence of clinical signs, and the lack of abnormalities on physical and neurological examinations, muscular disorders were ruled out. However, further examinations should be performed to evaluate muscle disorders.

## 5. Conclusion

This case report describes a suspected macro‐CK type 1 in a clinically healthy young cat, identified through CK isoenzyme and CK‐MB assay. Macro‐CK type 1 should be considered when persistent CK elevation is observed in asymptomatic cats. Although we could not perform due to the owner′s request, advanced diagnostic modalities such as CT, MRI, or electromyography may be valuable complementary tools. Future studies and feline‐specific assays are needed to establish definitive diagnostic criteria for macro‐CK type 1.

## Funding

No funding was received for this manuscript.

## Conflicts of Interest

The authors declare no conflicts of interest.

## Data Availability

All data generated during the study are included in this article.

## References

[1] Baird M. F. , Graham S. M. , Baker J. S. , and Bickerstaff G. F. , Creatine-Kinase- and Exercise-Related Muscle Damage Implications for Muscle Performance and Recovery, Journal of Nutrition and Metabolism. (2012) 2012, 10.1155/2012/960363, 2-s2.0-84866482920, 960363, 22288008. 22288008 PMC3263635

[2] Dawson D. M. , Eppenberger H. M. , and Kaplan N. O. , Creatine Kinase: Evidence for a Dimeric Structure, Biochemical and Biophysical Research Communications. (1965) 21, no. 4, 346–353, 10.1016/0006-291x(65)90200-7, 2-s2.0-0013852794. 5865501

[3] Kanemitsu F. and Okigaki T. , Creatine Kinase Isoenzymes, Journal of Chromatography A. (1988) 429, 399–417, 10.1016/S0378-4347(00)83880-3, 2-s2.0-0023719808. 3062028

[4] Horino T. , Ichii O. , Ode-Hamada K. , and Terada Y. , Macromolecular Creatine Kinase Type 1 in Immune-Mediated Necrotizing Myopathy, QJM: An International Journal of Medicine. (2017) 110, no. 9, 593–594, 10.1093/qjmed/hcx113, 2-s2.0-85038243876, 28633310. 28633310

[5] Giampietro O. , Clerico A. , Buzzigoli G. , Lucchetti L. , Boni C. , Del Chicca M. G. , and Mariani G. , Macro Serum CK-BB in a Woman With Severe Primary Hypothyroidism, American Journal of the Medical Sciences. (1985) 289, no. 4, 160–163, 10.1097/00000441-198504000-00006, 2-s2.0-0022236143, 3985049. 3985049

[6] Galarraga B. , Sinclair D. , Fahie-Wilson M. N. , McCrae F. C. , Hull R. G. , and Ledingham J. M. , A Rare but Important Cause for a Raised Serum Creatine Kinase Concentration: Two Case Reports and a Literature Review, Rheumatology. (2003) 42, no. 1, 186–188, 10.1093/rheumatology/keg039, 2-s2.0-0037238688, 12509637. 12509637

[7] Liu C. Y. , Lai Y. C. , Wu Y. C. , Tzeng C. H. , and Lee S. D. , Macroenzyme Creatine Kinase in the Era of Modern Laboratory Medicine, Journal of the Chinese Medical Association. (2010) 73, no. 1, 35–39, 10.1016/s1726-4901(10)70019-8, 2-s2.0-77951017178, 20103489. 20103489

[8] Silestri N. J. and Wolfe G. I. , Asymptomatic/Pauci-Symptomatic Creatine Kinase Elevations (Hyperckemia), Muscle Nerve. (2013) 47, no. 6, 805–815, 10.1002/mus.23755, 2-s2.0-84878351629, 23625835. 23625835

[9] Lin Y. J. , Chang L. W. , and Hung S. C. , Macro-Ck Type 2 Syndrome in Prostate Adenocarcinoma: Case Report and Review Article, Case Reports in Urology. (2021) 39, 101805, 10.1016/j.eucr.2021.101805, 34430214. PMC8365386 34430214

[10] Paltrinieri S. , Cazzaniga S. , da Cunha N. P. , and Giordano A. , Electrophoretic Fractionation of Creatine Kinase Isoenzymes and Macroenzymes in Clinically Healthy Dogs and Cats and Preliminary Evaluation in Central Neurologic Disease, Veterinary Clinical Pathology. (2010) 39, no. 3, 329–336, 10.1111/j.1939-165X.2010.00242.x, 2-s2.0-78149331703, 20698943. 20698943 PMC7169260

[11] Roberts R. , Gowda K. S. , Ludbrook P. A. , and Sobel B. E. , Specificity of Elevated Serum MB Creatine Phosphokinase Activity in the Diagnosis of Acute Myocardial Infarction, American Journal of Cardiology. (1975) 36, no. 4, 433–437, 10.1016/0002-9149(75)90890-5, 2-s2.0-0016777907. 1190047

[12] Lee K. N. , Csako G. , Bernhardt P. , and Elin R. J. , Relevance of Macro Creatine Kinase Type 1 and Type 2 Isoenzymes to Laboratory and Clinical Data, Clinical Chemistry. (1994) 40, no. 7, 1278–1283, 10.1093/clinchem/40.7.1278. 8013099

[13] Paltrinieri S. , Pintore L. , Balducci F. , Giordano A. , Costabile A. , and Bernardini M. , Serum Creatine Kinase Isoenzymes and Macroenzymes in Dogs With Different Neurologic Diseases, Veterinary Clinical Pathology. (2017) 46, no. 1, 91–99, 10.1111/vcp.12443, 2-s2.0-85014920762, 28085207. 28085207

